# Supplementary material for: Antibiotic-induced population fluctuations and stochastic clearance of bacteria
Source: eLife. 2018 Mar 6;7:e32976. doi: 10.7554/eLife.32976 (PMC5847335; doi:10.7554/eLife.32976)
Supplement: Supplementary file 1. — Source data: Source data for all figures [file elife-32976-supp1.docx]

| **Antibiotic** | **Minimum Inhibitory Concentration (MIC)** |
| --- | --- |
| Tetracycline* | 1.3 µg/ml |
| Erythromycin* | 65 µg/ml |
| Chloramphenicol* | 4.5 µg/ml |
| Thiolutin* | 6.0 µg/ml |
| Kanamycin** | 6 µg/ml |
| Ciprofloxacin** | 0.03 µg/ml |
| 6-APA** | 15 µg/ml |
| Streptomycin** | 12 µg/ml |
| Ofloxacin** | 0.096 µg/ml |
| Rifampicin** | 10 µg/ml |
| Cefsulodin** | 30 µg/ml |
| Vancomycin** | 128 µg/ml |

*bacteriostatic, **bactericidal
